# Guy's Hospital and the Southwark Guardians

**Published:** 1904-03-12

**Authors:** 


### GUY'S HOSPITAL AND THE SOUTHWARK GUARDIANS.

We take the following statement from the *Times* of 5th inst: Recently the Southwark guardians complained of the action of the authorities of Guy's Hospital in sending to their workhouse a man named Reid, who was suffering from stricture. Sent by his private doctor to the hospital to be operated upon, Reid, it was alleged, was rendered worse by an unsuccessful operation, and, instead of the young house surgeon's calling upon his superiors to remedy the case, he packed the man off to St. George's Workhouse, where there is no convenience for carrying out operations. Here Reid was received at 2 o'clock in the morning unconscious, and, although relieved by the medical officer of the institution, was too ill to allow of his removal to the infirmary. The guardians, not receiving a satisfactory reply to a complaint which they addressed to the house committee of the hospital, decided, on the motion of the Mayor of Southwark, to send particulars of the case and of two other recent similar ones to the King Edward's Hospital Fund Committee. The guardians have just received a letter from the committee, stating that they deeply regretted the difference that had arisen between the board and the authorities of Guy's, but adding that the committee had no power to hold an official inquiry into the circumstances. The matters referred to would, however, so far as they bore on the administration of the hospital, have their most careful consideration. Mr. Stranks, the chairman of the workhouse committee, said yesterday that the guardians were quite satisfied with the result of their action, for it had fully answered their purpose. Although there had not been, and would not be, any official declaration, he knew that the hospital had changed their policy and had taken precautions to see that no such "scandals" would recur. The guardians had no ill-feeling against Guy's, which was doing a splendid work, and he believed that the relations between both authorities would continue friendly, and that there would be no further cause for complaint.

